# Evidence for the High Importance of Co-Morbid Factors in *HFE* C282Y/H63D Patients Cared by Phlebotomies: Results from an Observational Prospective Study

**DOI:** 10.1371/journal.pone.0081128

**Published:** 2013-12-05

**Authors:** Philippe Saliou, Gérald Le Gac, Anne-Yvonne Mercier, Brigitte Chanu, Paul Guéguen, Marie-Christine Mérour, Isabelle Gourlaouen, Sandrine Autret, Cédric Le Maréchal, Karen Rouault, Jean-Baptiste Nousbaum, Claude Férec, Virginie Scotet

**Affiliations:** 1 Inserm, UMR 1078, Brest, France; 2 Université de Bretagne Occidentale, Brest, France; 3 Etablissement Français du Sang – Bretagne, Brest, France; 4 CHRU Brest, Hôpital Morvan, Laboratoire d’hygiène et de santé publique, Brest, France; 5 CHRU Brest, Hôpital Morvan, Laboratoire de Génétique Moléculaire et d’Histocompatibilité, Brest, France; 6 CHRU Brest, Hôpital La Cavale Blanche, Service d’Hépato-gastroentérologie, Brest, France; Penn State Hershey Medical Center, United States of America

## Abstract

Despite type I haemochromatosis (HC) is mainly associated with the *HFE* C282Y/C282Y genotype, a second genotype -C282Y/H63D- has mostly been described in other patients. Its association with HC, apart from any associated co-morbid factors, remains unclear and complex to interpret for physicians. This study assesses the weight of this genotype and the role of co-morbid factors in the occurrence of iron overload. This prospective study included the C282Y/C282Y (n = 172) and C282Y/H63D (n = 58) patients enrolled in a phlebotomy program between 2004 and 2007 in a blood centre of western Brittany (Brest, France), where HC is frequent. We compared prevalence of these two genotypes, as well as patients’ profile regarding degree of iron overload and prevalence of co-morbid factors. First, we confirmed the obvious deficit of C282Y/H63D compound heterozygotes among patients cared by phlebotomies. This genotype was 3.0 times less frequent than the C282Y/C282Y genotype among those patients (18.9% *vs.* 56.0%) whereas it was 4.9 times more frequent in the general population (4.3% *vs.* 0.9%; p<0.0001). Despite a similar level of hyperferritinaemia, the C282Y/H63D patients who came to medical attention had a milder plasma iron overload, reflected by a lower transferrin saturation median (52.0% *vs.* 84.0%; p<0.0001). They also exhibited more frequently co-morbid factors, as heavy drinking (26.0% *vs.* 13.9%; p = 0.0454), overweight (66.7% *vs.* 39.4%; p = 0.0005) or both (21.3% *vs.* 2.6%; p<0.0001). Ultimately, they required a lower amount of iron removed to reach depletion (2.1 *vs.* 3.4 g; p<0.0001), clearly reflecting their lower tissue iron. This study confirms that H63D is a discrete genetic susceptibility factor whose expression is most visible in association with other co-factors. It highlights the importance of searching for co-morbidities in these diagnostic situations and of providing lifestyle and dietary advice.

## Introduction

Haemochromatosis (HC; EASL consensus conference 2000 [1]) refers to a group of inborn errors of iron metabolism characterized by increased absorption of dietary iron and rapid iron release from macrophages. If not diagnosed and treated early, iron loading can lead to tissue damages and severe clinical complications, including cirrhosis, hepatocellular carcinoma and cardiomyopathy [2]. The treatement relies on regular phlebotomies that are, in our country, mostly managed in blood centres.

The best-known and predominant form of HC is an adult-onset autosomal recessive condition mostly associated with the *HFE* C282Y homozygous genotype (p.[Cys282Tyr]+[Cys282Tyr] according to the recommandations of the Human Genome Variation Society - http://www.hgvs.org). This genotype is carried by approximately 1 person in 200 in Northern European populations [3]. However, not all of them develop clinical features of HC. Full expression of iron overload in people with this genotype depends on age and gender, plus the complex interplay of environmental factors and modifier genes [4]–[5].

A second *HFE* variant – H63D (p.[His63Asp]) – has been identified in HC patients but its relation with disease remains unclear. An important enrichment of this variant has initially been reported on the non-C282Y alleles of HC patients [6], and it has subsequently been argued that the C282Y/H63D compound heterozygous genotype (p.[Cys282Tyr]+[His63Asp]), but not the H63D homozygous genotype (p.[His63Asp]+[His63Asp]), increases the disease risk [7]–[8]. Although relating to a very low penetrance [4], [9]–[11], detection of C282Y/H63D compound heterozygotes was therefore recommended in the outline of most (if not all) diagnostic strategies in patients with suspected HC. In recent years, the perception of the H63D variant, which is highly prevalent in Caucasians (average allelic frequency of 14% [12]), has moved toward the notion of a susceptibility factor *i.e.* a variant that has no impact on disease if considered in isolation. It has thus been reported that most of compound heterozygotes with iron overload usually have associated co-morbid factors (such as heavy drinking and obesity) [13].

It is therefore probable that co-morbid factors are more frequent in C282Y/H63D heterozygotes diagnosed than in C282Y/C282Y homozygotes because of an hyperferritinemia, and that hyperferritinemia observed in most of C282Y/H63D compound heterozygotes does not totally match with the classical iron overload hallmarks of HC. If checked, this assumption will emphasize the importance of acting on co-morbid factors during care.

The aim of our study was to assess the profile of the C282Y/H63D patients treated by phlebotomies and to investigate the role of the co-morbid factors in the phenotypic expression. For that, we analysed a cohort of patients cared in a blood centre of western Brittany (France), where HC is particularly common [14]. We clearly show here that co-morbid factors (such as alcohol abuse and overweight) are significantly more prevalent in C282Y/H63D patients who come to medical attention than in C282Y/C282Y patients.

## Materials and Methods

### Ethics Statement

The study protocol was approved by our local ethical committee (University Hospital of Brest). Written informed consent was obtained from each patient included in the study.

### Study Population

The present study was conducted from the cohort of patients enrolled in a phlebotomy program in a blood centre of western Brittany (Brest, France) between January 1^st^ 2004 and December 31^st^ 2007 (year from which the H63D was not systematically searched for in our laboratory) (n = 325). These patients were referred to the blood centre by general practitioners or gastroenterologists, for the achievement of phlebotomies because they had elevated serum ferritin (≥300 µg/L for males or ≥200 µg/L for females).

Our attention was mainly focused on patients carrying the *HFE* C282Y/C282Y or C282Y/H63D genotypes (n = 172 and n = 58, respectively).

### Case Report Form

The study relies on the data collected in a clinical questionnaire, which is completed by a referral physician at entrance in the phlebotomy program. As previously described [15], this questionnaire yields information on socio-demographic characteristics (gender, age, age at diagnosis, etc), lifestyle factors (weight, height, alcohol intake, etc), biological parameters (including transferrin saturation (TS) and serum ferritin (SF)), and clinical features associated at the time of diagnosis (asthenia, melanodermia, hepatomegaly, diabetes, cirrhosis, etc).

Data related to the treatment (number and volume of phlebotomies) are recorded thereafter, when depletion is reached. These data enable to assess the amount of iron removed (AIR; in grams) needed for normalising iron stores (*i.e.* decreasing SF below the threshold of 50 µg/L).

Regarding lifestyle factors, daily consumption of alcohol was estimated by asking patients to state the number of glasses of different kinds of alcohol (beer, wine, fortified wine, hard liquor) they drank each day. In our area, people mainly drink red wine (especially during meals) and, in second line, beer. Daily consumption corresponded to the sum of the numbers of glasses declared. Whatever the liquor, a standard drink (representing one unit of alcohol) contains the same amount of pure alcohol (ethanol), namely 10 grams in our country. Thereby, in France, a glass of 25 cl of beer (at 5°), of 10 cl of wine (12°), of 7 cl of fortified wine (18°) or of 3 cl of spirits (40°) contains about 10 grams of pure alcohol. In accordance with the WHO definition, alcohol intake was considered abusive when consumption exceeded 3 glasses per day for men (*i.e.* 21 glasses per week) and 2 glasses per day for women (*i.e.* 14 glasses per week). The second studied co-morbid factor – overweight – was defined by a body mass index (BMI) ≥25 kg/m^2^ and obesity by a BMI ≥30 kg/m^2^.

### Measurement of Iron Markers and Genetic Analysis

Iron markers were measured by standard biochemical methods. The C282Y and H63D variants were studied using the 5′- nuclease allelic discrimination method or TaqMan method (Applied Biosystems, Foster City, CA).

### Statistical Analysis

Statistical analysis was carried out using the SAS software (version 9.02; SAS Institute Inc, Cary, NC). All tests were performed two-sided, and a p-value less than 5% was considered significant.

First, we first described the socio-demographic characteristics of the population under study. Then, we assessed the impact of the C282Y/H63D genotype and proceeded, for that, in three steps:

1. We quantified the deficit of C282Y/H63D compound heterozygotes observed among patients who come to medical attention. For that, we determined the prevalence of the C282Y/H63D and C282Y/C282Y genotypes in the study population (*i.e.* among patients enrolled in a phlebotomy programme) and compared it to that observed in the general population (*i.e.* in a sample of 797 blood donors from our area).
2. We compared the nature and severity of iron overload (assessed by TS, SF and AIR) in C282Y/H63D and C282Y/C282Y patients who came to medical attention. We performed linear regression analysis to investigate the influence of the genotype on the level of iron overload. We performed logarithmic transformation (log_10_) of the dependant variables (*i.e.* TS, SF and AIR) in order to normalise their distribution that were highly skewed. With log_10_ transformation, the estimated regression coefficients (β) corresponded to a 10^β^ time change in the dependant variable per unit increase in the explanatory variables. After univariate analyses, multivariate models were fitted to enable adjustment on potential confounding factors, such as gender, age at diagnosis, alcohol intake and overweight. Two-by-two interactions were also tested.
3. We examined whether the C282Y/H63D patients had more commonly co-morbid factors known to cause hyperferritinemia (alcohol abuse and overweight). We therefore compared the frequency of these factors in the two genotypic groups.

## Results

### Description of the Study Population

Over the study period, 325 patients were enrolled in a phlebotomy program at the blood centre of Brest. *HFE* genotypic data were complete for 306 of them (94.2%). Among these patients, 172 were C282Y/C282Y homozygotes and 58 C282Y/H63D compound heterozygotes. The group of C282Y/H63D patients cared by phlebotomies tended to have a higher proportion of men (70.7% *vs.* 56.4%; p = 0.0547) and was on average diagnosed later (56.9 *vs.* 49.3 y.; p<0.0001) than the group of C282Y/C282Y patients.

### Frequency of the Different *HFE* Genotypes

One way to confirm the milder effect of the C282Y/H63D genotype was to compare the prevalence of the various *HFE* genotypes observed respectively in the group of patients treated by phlebotomies and in the general population (Table 1).

**Table 1:** Frequency of *HFE* genotypes observed in the study population (*i.e.* among patients enrolled in a phlebotomy programme) and in the general population.

*HFE* genotype	Study population		General population	
	n	%	n	%
C282Y/C282Y	172	56.2%	7	0.9%
C282Y/H63D	58	18.9%	34	4.3%
H63D/H63D	18	5.9%	15	1.9%
C282Y/wt*	11	3.6%	105	13.1%
H63D/wt*	25	8.2%	164	20.6%
wt*/wt*	22	7.2%	472	59.2%
Total	306	100.0%	797	100.0%

*wt: wild type.

The C282Y/H63D genotype appears relatively common in our population. It is carried by 1 individual in 23 (*i.e.* 4.3%) whereas the C282Y/C282Y genotype is found in 1 individual in 110 (*i.e.* 0.9%). The H63D and C282Y allele frequencies are 14.3% (95% confidence interval (CI), 12.6**–**16.0) and 9.6% (95% CI, 8.2**–**11.0), respectively.

By focusing on the C282Y/C282Y and C282Y/H63D genotypes, we observed that, in the general population, the C282Y/H63D genotype was 4.9 times more frequent than the C282Y/C282Y genotype (4.3% *vs.* 0.9%). However, this genotype appeared 3.0 times less frequent than the main genotype among patients enrolled in a phlebotomy program (18.9% *vs.* 56.0%). This confirmed the obvious deficit of C282Y/H63D compound heterozygotes among patients cared by phlebotomies. We estimated that they were 14.4 times less frequently observed (OR, 0.07; 95% CI, 0.03**–**0.17; p<0.0001). As expected, most of the patients carrying this genotype did not come to medical attention because of their mild, if any, iron overload.

### Degree of Iron Overload

We then compared the severity of iron overload in C282Y/H63D and C282Y/C282Y patients treated by phlebotomies. As illustrated in Table 2, the C282Y/H63D genotype was clearly associated with a milder iron overload. The amount of plasma iron was milder in these patients, as reflected by a lower median TS (52.0% *vs.* 84.0% for the C282Y/C282Y patients; p<0.0001). One third of them (32.7%) had a TS value <45% and did not meet the diagnostic criteria of HC. Only a very small proportion of these patients had a TS value ≥80% (3.9% *vs.* 58.0%; p<0.0001). It should be noted that TS was not determined in 28 patients (22 homozygotes and 6 compound heterozygotes) that were taken in care at the end of 2007 when the non-payment of this examination by our health insurance was introduced.

**Table 2:** Comparison of serum markers in C282Y/H63D and C282Y/C282Y patients who were enrolled in a phelebotomy program at the blood centre of Brest (western Brittany, France) between 2004 and 2007.

	C282Y/H63D	C282Y/C282Y	p
	Median (Q1–Q3)*	Median (Q1–Q3)*	
**Transferrin saturation (%)**	**52.0 (40.0–60.5)**	**84.0 (67.0–92.0)**	**<0.0001**
Males	57.0 (47.0–69.0)	87.5 (75.5–94.5)	<0.0001
Females	42.0 (37.0–51.0)	73.5 (64.0–89.0)	<0.0001
**Amount of iron removed (g)**	**2.1 (1.3–2.8)**	**3.4 (1.9–6.0)**	**<0.0001**
Males	2.3 (1.7–2.9)	5.2 (3.2–7.0)	<0.0001
Females	1.3 (1.0–2.2)	2.1 (1.5–3.5)	0.0132
**Serum ferritin (ng/ml)**	**529.0 (414.0–937.0)**	**692.0 (392.5–1293.5)**	**0.1003**
Males	659.0 (448.0–1072.0)	927.0 (649.0–1700.0)	0.0032
Females	422.0 (300.0–665.0)	414.0 (272.0–733.0)	0.7913

*Q1: first quartile; Q3: third quartile.

Similar findings were obtained with the AIR by phlebotomies, which is a good marker of the iron burden. This marker was indeed significantly lower in the C282Y/H63D patients cared by phlebotomies than in the C282Y/C282Y ones (median: 2.1 *vs.* 3.4 g; p<0.0001).

The milder role of the C282Y/H63D genotype on the level of iron overload was confirmed by linear regression analysis (Table 3). The multivariate analysis showed that, after adjustment for gender, age at diagnosis, alcohol intake and overweight, TS was 1.56 fold lower in the C282Y/H63D patients (10^β^, 0.64; 95% CI, 0.58**–**0.71; p<0.0001), and AIR 1.29 fold lower (10^β^, 0.78; 95% CI, 0.71**–**0.85; p<0.0001).

**Table 3:** Parameter estimates of the linear regression model testing the effect of genotype on the level of serum markers, after adjustment on covariables.

Variables		10^β^	(95% CI)	p
**Transferrin saturation**				
Genotype (ref: C282Y/C282Y)		**0.64**	**(0.58–0.71)**	**<0.0001**
Gender (ref: women)		1.15	(1.07–1.25)	0.0005
Age at diagnosis (ref: <40 y.)				
[40 y. –50 y.]		1.07	(0.95–1.20)	0.2731
[50 y. –60 y.]		1.08	(0.96–1.21)	0.1790
≥60 y.		1.09	(0.95–1.24)	0.2235
Heavy drinking		1.05	(0.95–1.16)	0.3273
Overweight		0.92	(0.85–0.99)	0.0353
**Amount of iron removed**				
Genotype (ref: C282Y/C282Y)		**0.78**	**(0.71–0.85)**	**<0.0001**
Gender (ref: women)		1.11	(1.03–1.20)	0.0082
Age at diagnosis (ref: <40 y.)				
[40 y. –50 y.]		0.96	(0.85–1.07)	0.4472
[50 y. –60 y.]		1.00	(0.89–1.12)	0.9730
≥60 y.		0.96	(0.84–1.09)	0.5005
Heavy drinking		1.11	(1.00–1.22)	0.0441
Overweight		0.96	(0.89–1.04)	0.3459
**Serum ferritin**				
Genotype (ref: C282Y/C282Y)		**0.68**	**(0.55–0.85)**	**0.0009**
Gender (ref: women)		1.89	(1.58–2.27)	<0.0001
Age at diagnosis (ref: <40 y.)				
[40 y. –50 y.]		1.16	(0.89–1.53)	0.2768
[50 y. –60 y.]		1.36	(1.04–1.77)	0.0250
≥60 y.		1.30	(0.97–1.75)	0.0799
Heavy drinking		1.76	(1.39–2.22)	<0.0001
Overweight		1.12	(0.94–1.34)	0.2130

In contrast, the level of hyperferritinaemia appeared similar between the two genotypic groups (median, 529.0 *vs.* 692.0 mg/L; p = 0.1003). Nevertheless, after adjustment on potential confounders, the role of the genotype on SF became significant. This marker was 1.47 fold lower in the C282Y/H63D patients (10^β^, 0.68; 95% CI, 0.55**–**0.85; p = 0.0009).

### Frequency of Co-morbid Factors

Finally, we checked whether the C282Y/H63D patients who came to medical attention had more commonly co-morbid factors that could explain their hyperferritinemia. Data on alcohol intake and on body mass index were not documented for 15 (6.5%) and 16 (7.0%) patients respectively, but the frequency of these missing values did not differ according to genotype (p = 0.0917).

**Figure 1:**
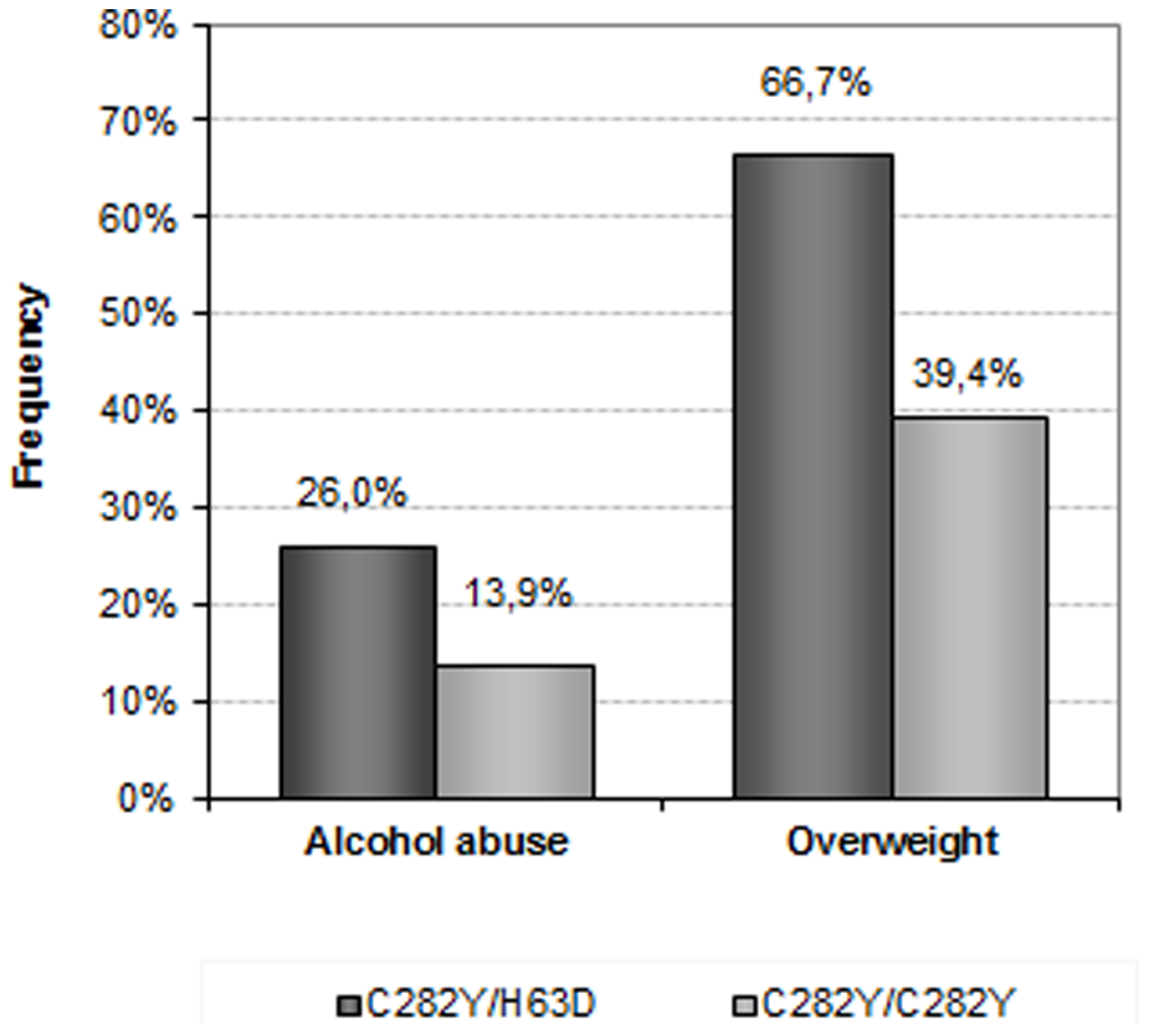
Frequency of co-morbid factors in C282Y/H63D and C282Y/C282Y patients who were enrolled in a phlebotomy program at the blood centre of Brest (western Brittany, France) between 2004 and 2007.

We observed that the co-morbid factors were significantly more frequent in the group of C282Y/H63D patients (**Fig. 1**). Heavy drinking was thus reported by 26.0% of those patients (*vs.* 13.9% of the C282Y/C282Y homozygotes; p = 0.0454), and overweight was observed in 66.7% of them (*vs.* 39.4%; p = 0.0005). Similar results tended to be observed for obesity (20.4% *vs.* 10.6%; p = 0.0663).

In summary, three quarters of the C282Y/H63D patients had at least one of these co-morbid factors (75.0% *vs.* 51.3% of the C282Y/C282Y homozygotes; p = 0.0026), and more than one fifth of them had the two factors (21.3% *vs.* 2.6%; p<0.0001). One can also note that all the C282Y/H63D patients having a SF ≥1000 µg/L had at least one of these lifestyle factors (*vs.* 63.0% for the C282Y/C282Y patients; p = 0.0116).

## Discussion/Conclusions

The present study confirms that the C282Y/H63D genotype is clearly associated with a milder iron overload. Patients carrying this genotype that are cared by phlebotomies have both a milder amount of plasma iron (reflected by lower TS) and of tissue iron (reflected by lower AIR required to reach depletion). These patients also exhibit more frequently co-morbid factors (as heavy drinking and/or overweight) that may alone explain their hyperferritinemia (whose level did not seem different from that of the C282Y/C282Y patients). This study confirms therefore the modest weight of the C282Y/H63D genotype and the major role of co-morbid factors in the occurrence of biological expression of HC.

The lack of C282Y/H63D genotypes among patients enrolled in a phlebotomy program is an additional argument reflecting the modest role of this genotype in the occurrence of iron overload. Indeed, we have shown here that the C282Y/H63D genotype was 3.0 times less frequent than the C282Y/C282Y genotype among patients enrolled in a phlebotomy program whereas it was 4.9 times more frequent in the general population. As expected, most of the patients carrying this genotype do not come to medical attention because of their mild, if any, iron overload.

Beyond these results, it is also important to keep in mind that this C282Y/H63D genotype has only rarely been reported in a family context. In our experience, we have never seen a family with two or more sibling carrier of the genotype C282Y/H63D that present an iron overload.

It should be noted that, in our cohort, less than one third of patients had a SF ≥1000 µg/L (30.9% *i.e.* 33.7% of the C282Y/C282Y patients and 22.4% of the C282Y/H63D patients). The discovery of the *HFE* gene in 1996 and the availability of the DNA test have greatly changed the clinical presentation of the disease [16], and it is now rare to diagnose severe forms, associating darkened skin, diabetes and cirrhosis. Diagnosis is made at an earlier stage, particularly in areas like ours, where the prevalence of the disease is high and where physicians are very aware of the disease.

The originality of our study was to investigate the role of both *HFE* genotype and co-morbid factors (alcohol, overweight) on the disease expression, this from a cohort of patients cared by phlebotomies. Our study focused on a cohort of treated patients while, so far, most studies have been conducted in the general population and attempted to estimate the penetrance of the C282Y/H63D genotype. Another strength of our study, which was performed in an area where HC is particularly common, was its ability to assess the influence of co-morbid factors. We clearly showed here that alcohol abuse and overweight were significantly more prevalent in C282Y/H63D patients in comparison to C282Y/C282Y patients.

One limitation of our study was our inability to determine the true prevalence of the metabolic syndrome, which combines the presence of several metabolic abnormalities (abdominal obesity, low HDL cholesterol, high triglycerides, glucose intolerance, hypertension) [17]. It is obvious that a part of the C282Y/H63D patients may have such a syndrome, which is known to be one of the main causes of hyperferritinemia [18]. Unfortunately, until now, our questionnaire did not collect all the information needed to clearly define it (waist circumference is not measured, making it impossible to assess the presence of abdominal obesity). Nevertheless, in absence of this indicator, we focused our attention on overweight and obesity. Furthermore, we compared the frequency of overweight observed in each genotypic group with that observed in the French population by referring to a nationwide survey on overweight (Obepi) [19]. We noticed that, if the frequency of overweight was similar to that of the general population in our cohort of C282Y homozygotes (39.4% *vs.* 46.4%; p = 0.2140), it was significantly higher in compound heterozygotes (67.3% *vs.* 46.4%; p = 0.0340). This confirms the role of co-morbid factors in the occurrence of iron overload in the C282Y/H63D patients.

Another limitation was that the possible existence of modifier genes. We re-sequenced the five genes associated with haemochromatosis (*HFE*, *HAMP*, *HJV*, *TFR2*, *SLC40A1*) in 23 C282Y/H63D patients for whom DNA was available (knowing that the initialanalysis of *HFE* gene was not carried out by our laboratory for all patients). These genes are involved in the same physiological pathway that controls production of hepcidin to fit body iron needs and avoid iron accumulation in tissues. No variation that could influence phenotypes observed in the 23 C282Y/H63D patients has been detected; only synonymous variants or common variations with no physiological consequence were observed. We are aware that rare variants in non-*HFE* genes can influence iron burden in C282Y/H63D patients. However we believe that they can be neglected in this study and that environmental factors are prominent in our patient sample.

Our findings are consistent with data reported in the literature illustrating that the perception of the H63D variant has moved, in recent years, toward the notion of susceptibility factor (*i.e.* a variant with no impact on disease if considered in isolation). In support of this, Gurrin *et al.* have evidenced, in a prospective and longitudinal study, that compound heterozygotes had an increased prevalence of elevated SF compared to subjects with neither the C282Y mutation nor the H63D variant, but that mean SF levels at baseline and follow-up remained within the normal range [11]. The authors were not able to determine whether C282Y/H63D heterozygotes with significant hyperferritinemia had co-morbid factors. In another study, Walsh *et al.* found that compound heterozygotes referred for clinical evaluation had higher iron indices than those identified though family screening. The authors extended their observations showing that of the C282Y/H63D subjects with a significant hyperferritinemia, all had known co-morbid factors such as heavy drinking or obesity [13]. It has also been showed that C282Y heterozygotes with a haemochromatosis pattern of hepatic iron overload are more likely to have a private mutation on the second *HFE* allele [20]–[21], while seconday causes of hyperferritinemia are frequent in C282Y heterozygotes without documented iron overload-related disease [21].

Regarding penetrance, a recent study, based on a cohort of 6020 Danish men aged 30 to 53 years has estimated the biological penetrance at 87.5% for the C282Y homozygotes and at only 9.1% for the C282Y/H63D compound heterozygotes (based on TS ≥50% and SF ≥300 µg/L) [10]. An other study recently performed in a Spanish Mediterranean population composed of 815 healthy subjects aged between 18 and 75 y. has reported that the C282Y/H63D genotype was associated with a 16.4% increase in TS and that it explained 37.7% of the variability observed in SF values [22].

Beyond this, we should also keep in mind that, at protein level, the H63D does not alter the HFE protein structure, in contrast to the C282Y that prevents interaction with beta_2_-microglobulin and presentation on cell surface [23]–[24].

In conclusion, our study provides evidence for the milder effect of the C282Y/H63D genotype in comparison to the C282Y/C282Y genotype by three ways: 1) through the lower frequency of this genotype among patients cared by phlebotomies than expected, 2) through the higher prevalence of associated co-morbid factors in these patients 3) and finally through the milder degree of iron overload in these patients.

Our results combined with data from the literature underline that the H63D variant, which is common in the general population, is a discrete genetic susceptibility factor whose expression is most visible in association with other co-factors such as alcohol abuse and/or overweight. Our study clearly highlights the importance of searching for co-morbidities in these diagnostic situations and of acting on these factors during care. In most cases, lifestyle and dietary advices should be sufficient to reduce the number of phlebotomies requested in those patients. This work should therefore contribute to improve the management of haemochromatosis patients.
